# Supplementary figures and images for: Modelling amoebic brain infection caused by Balamuthia mandrillaris using a human cerebral organoid
Source: PLoS Negl Trop Dis. 2024 Jun 20;18(6):e0012274. doi: 10.1371/journal.pntd.0012274 (PMC11218984; doi:10.1371/journal.pntd.0012274)

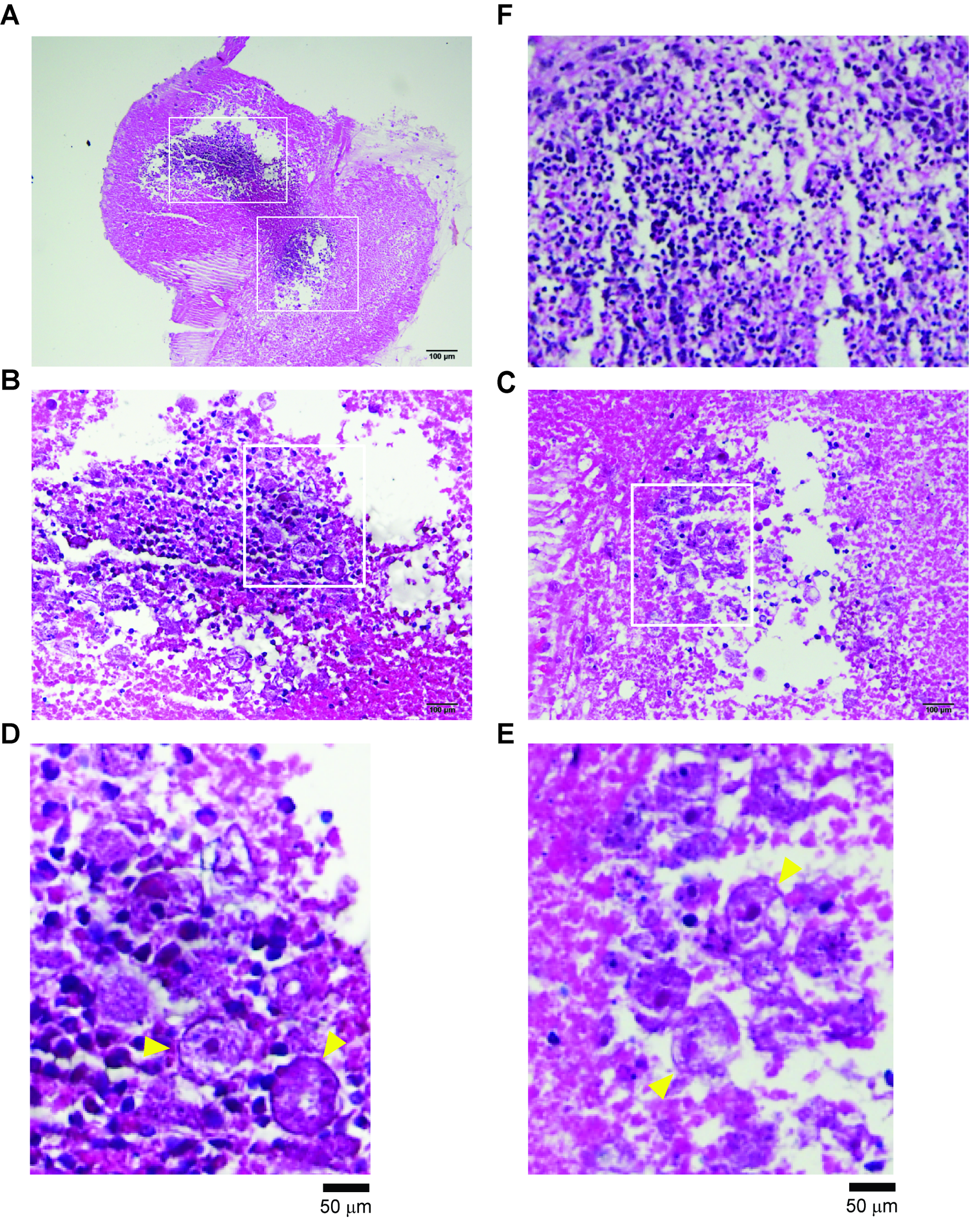

Supplement: S1 Fig — A 16-day coculture of B. mandrillaris trophozoites with human cerebral organoids was subjected to H&E staining. (A) Trophozoites of B. mandrillaris were observed inside the cerebral organoid, in which the cells formed clumps (white insets). (B and C) Higher magnification of insets in panel A shows trophozoites in the cell clump in the upper and lower inset of panel A, respectively. (D and E) Zoomed-in images of the insets in panels B and C, respectively. Yellow arrows indicate trophozoites with a round shape with nuclei in some cells. F. Noninfected cerebral organoids showed well-defined nuclei without cell clumps. Scale bars in panels A-C = 100 μm; scale bars in panels D-E = 50 μm. (TIF) [file pntd.0012274.s001.tif]

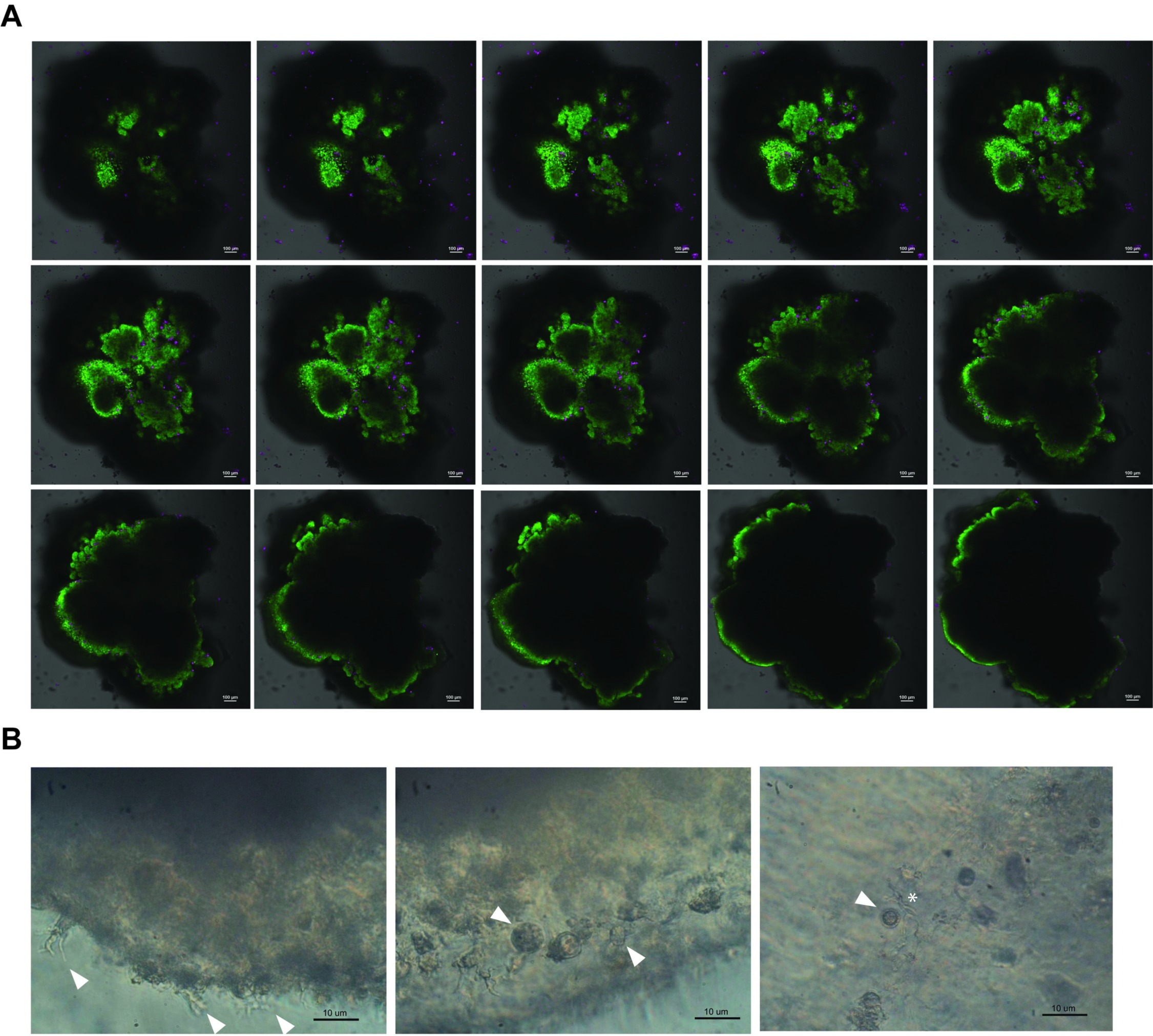

Supplement: S2 Fig — (A) Representative fluorescence images of cerebral organoids cocultured with trophozoites (scale bars, 200 μm). The cerebral organoids and the trophozoites were differentially labelled using the protein-binding CMFDA (green) and lipid-binding DiD (magenta) fluorescent markers, respectively. The 60-day cerebral organoid was incubated with the CMFDA for 1 hour followed by the co-culture with the DiD-labelled trophozoites. (B) Microscopy images of cerebral organoids cultured with trophozoites. On the surface, the trophozoites had multiple cytoplasm protrusions with active movement (arrowheads, left panel). Two days later, there were many trophozoites located at a small focal break in the cerebral organoids (arrowheads, right panel). Some trophozoites remained elongated in shape (asterisks, right panel), while most were oval (arrowheads, right panel). Scale bars, 100 μm. (TIF) [file pntd.0012274.s002.tif]

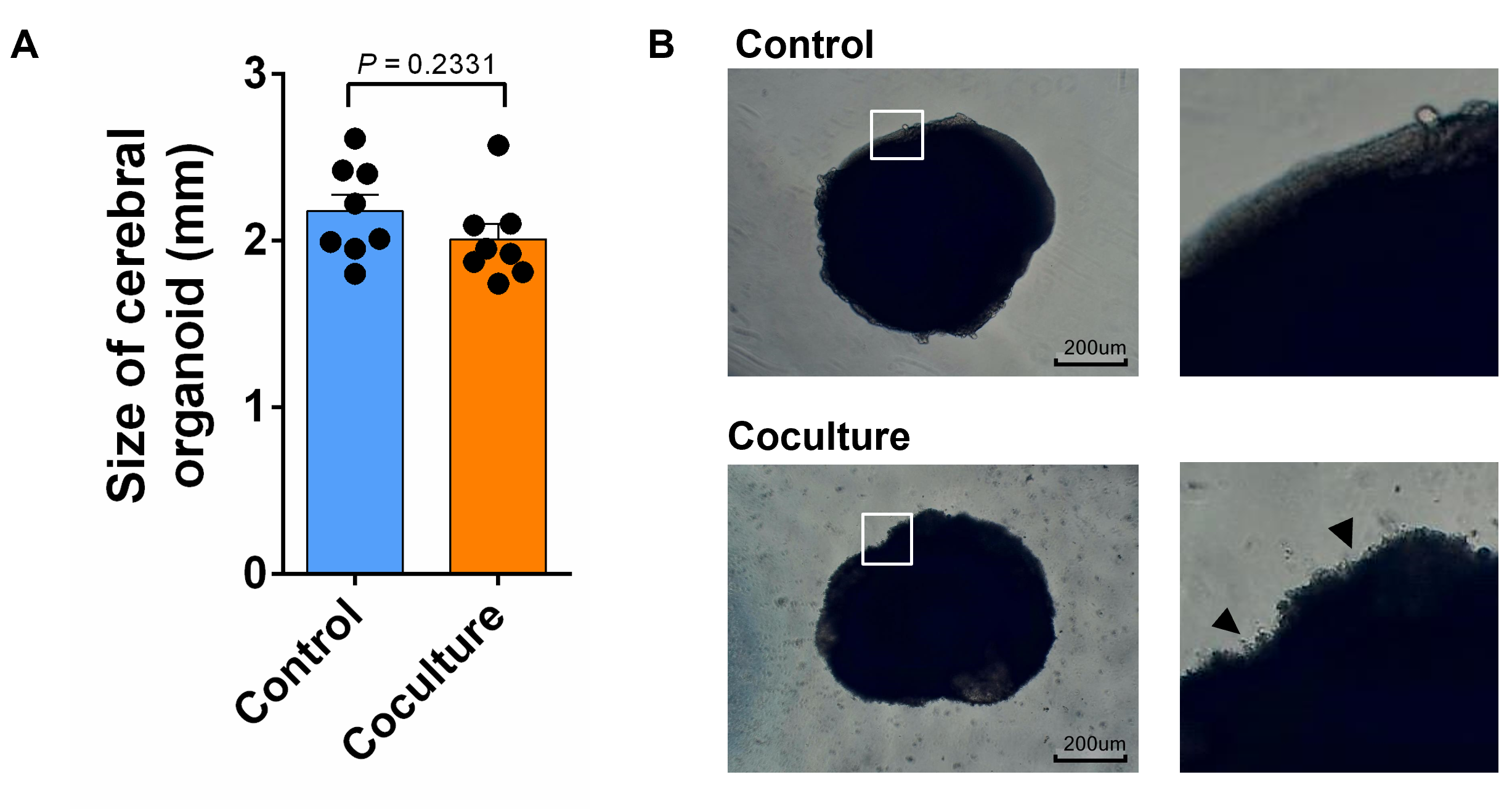

Supplement: S3 Fig — (A) The bar chart showing the diameter of human cerebral organoids and a X-day coculture of B. mandrillaris trophozoites with human cerebral organoids. Each dot is represented to the cerebral organoids (n = 8). The data are the mean ± SD. The statistical analysis was performed by an independent sample t test. (B) Representative images show the cerebral organoids in control and coculture experiments (scale bars, 200 μm). In the coculture with trophozoites, the outermost layer of the cerebral organoids is not smooth (arrowheads). (TIF) [file pntd.0012274.s003.tif]

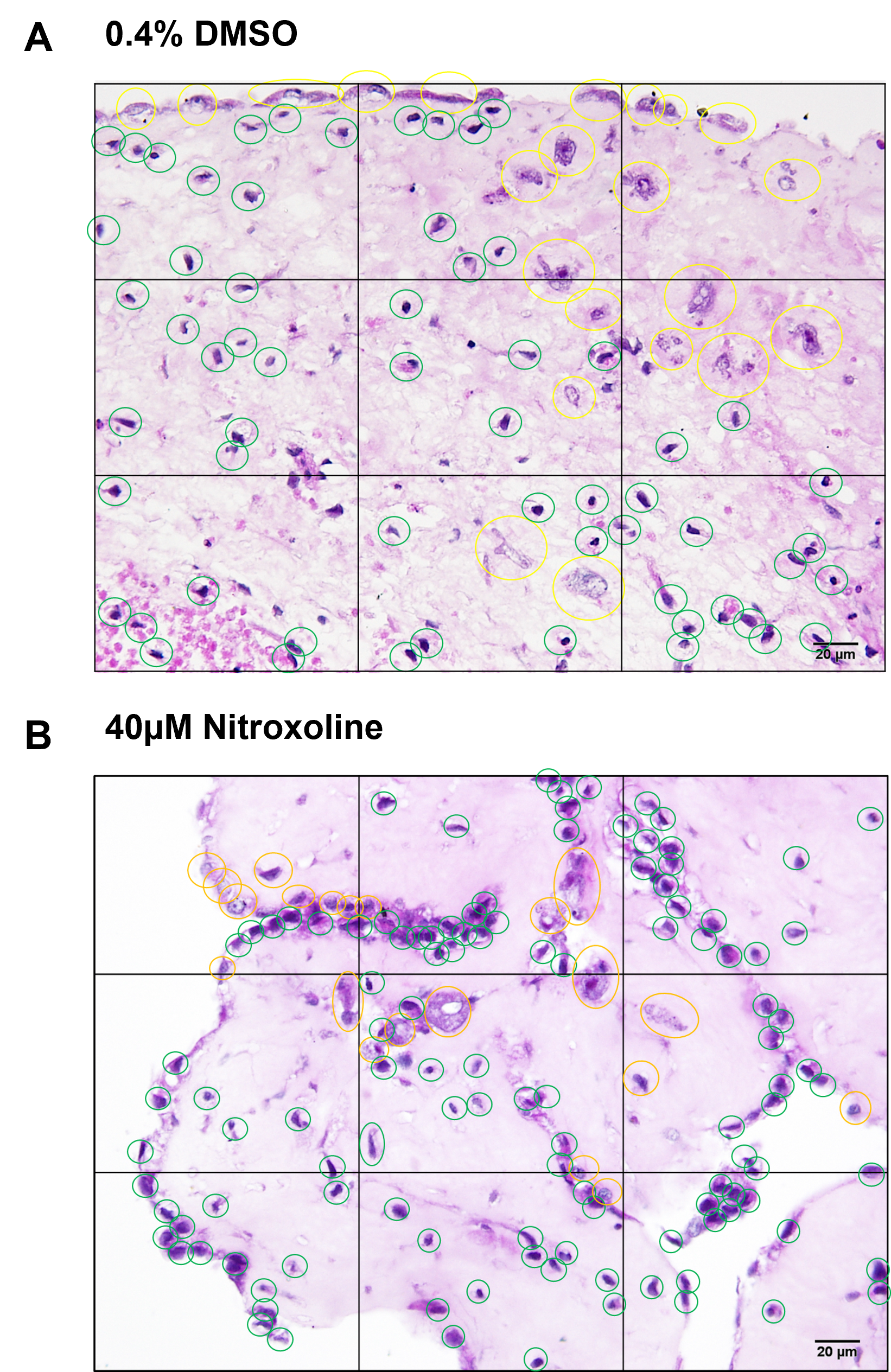

Supplement: S4 Fig — Three representative microscopic fields (magnification of 100X, objective lens) of coculture organoids with B. mandrillaris after DMSO and nitroxoline exposure were subjected to cell counting. The number of B. mandrillaris is enumerated based on polymorphic and round shape (yellow circle), while that of other cells is counted based on the nucleus stain (green circle). Three independent examiners and one pathological expert counted the number without knowing the sample types to avoid bias (n = 4). (TIF) [file pntd.0012274.s004.tif]

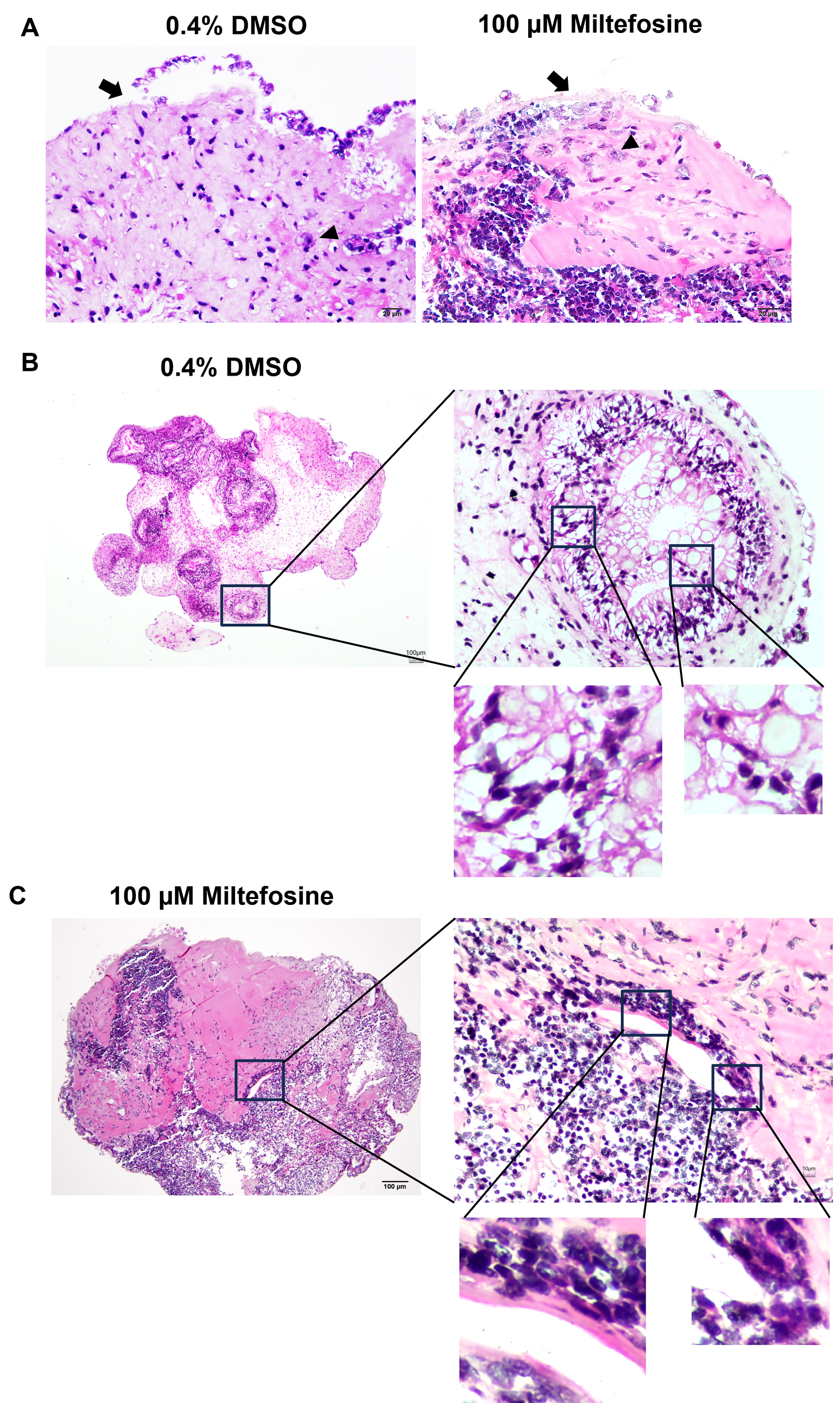

Supplement: S5 Fig — (A) Representative H&E staining images of cerebral organoids cocultured with trophozoites with and without 100 μM miltefosine (scale bars, 20 μm). In the DMSO-treated control (left panel), the trophozoites invaded the inner layer (arrowhead), while the meninge-like layer detached from the outermost layer (arrow). In the miltefosine-treated samples (right panel), the outermost layer of the cerebral organoids was attached by trophozoites (arrow). Some trophozoites had a large unstained space in the cytoplasm (arrowheads). (B) Representative H&E staining images of cerebral organoids in coculture with trophozoites and 0.4% DMSO. (C) The hollow inside the cerebral organoids after miltefosine exposure. There is no appearance of trophozoites in the hollow position. (TIF) [file pntd.0012274.s005.tif]
