# Supplementary material for: Modelling amoebic brain infection caused by Balamuthia mandrillaris using a human cerebral organoid
Source: PLoS Negl Trop Dis. 2024 Jun 20;18(6):e0012274. doi: 10.1371/journal.pntd.0012274 (PMC11218984; doi:10.1371/journal.pntd.0012274)
Supplement: Attachment — Submitted filename: Authorss Responses to Reviewers_270524.docx [file pntd.0012274.s011.docx]

May 27^th^, 2024

Dear Editors

We thank the reviewers for their constructive comments and generous suggestions on the submitted manuscript. According to the comments and suggestions, we have edited the revised manuscript to address their concerns.

In particular, we have responded point-by-point to the reviewer’s comments, with our responses immediately below the comment in blue color. The revised portions are in blue to track the changes in the revised manuscript. Also, we provide a clean version of the revised manuscript. Moreover, the English language in the revised manuscript was re-edited by American Journal Experts (the certificate is attached).

We believe that the revised manuscript is now suitable for publication in *PLOS Neglected Tropical Diseases.*

Yours sincerely,

Kasem Kulkeaw, PhD

**Reviewer Comments:**

**Reviewer #1:** This is an important paper that will help further research on a very neglected serious pathogen. The authors have succeeded in developing a useful and sophisticated model for studying host-amoeba interactions. However, I have one major and several minor comments that I think need to be considered.

1. My main criticism concerns the effect of nitroxoline on amoeba-induced organoid damage. First, it is not clearly described in the results, and second, there is only one image each with and without the drug, which is uninformative. More images are needed ideally some quantification.

**Response:**

We would like to thank you for the constructive feedback to improve the quality of the manuscript. Please find our response point-by-point below each comment.

To increase the informative data of the nitroxoline effect, we counted a number of the amoeba and human cells on the H&E-stained sections as follows.

1) Three representative microscopic fields (magnification of 100X, objective lens) of H&E-stained sections were subjected to cell counting.
2) The number of *B. mandrillaris* is enumerated based on polymorphic and round shape, while that of other cells is counted based on the nucleus stain.
3) Three independent examiners and one pathologist counted the number without knowing the sample types to avoid bias.
4) A bar graph was generated to show the number of amoebae per human cells (displayed as a percentage on the Y-axis)
5) Student t-test was used to compare the differences among the control and nitroxoline-treated groups.

There are the following changes in the revised manuscript.

- Bar graph showing percentage of amoeba as Fig. 2E
- Explanation of the quantitative data was in lines 359-362 (page 18).
- Additional microscopic images were in the S4 Fig.
- Statistical analysis is mentioned in lines 234-239 (page 12).

2. It is also unclear why the samples were analyzed 4 days after drug exposure, rather than 16 days. After a longer period of time, would the infection have developed regardless of the drug effect?

**Response:**

The experimental design initially followed the previous study of Laurie et al who first reported the effect of nitroxoline on the *B. mandrillaris* cocultured with human brain tissue explant. This study suggests the protective effect of nitroxoline in impeding cell destruction of human brain tissue. Thus, our work aims to confirm this protective effect in the context of human brain organoids.

Thanks for pointing out this unclear issue, we have noticed a lack of such an explanation in the result. Hence, we add an explanation about the purpose of the experiment, in which the coculture of brain organoids and *B. mandrillaris* was simultaneously treated with nitroxoline, and cell damage was assessed after 4 days of drug exposure (lines 350-354 on page 17).

Secondly, it is a very interesting question about cellular damage of brain organoids after drug exposure for longer than 4 days in the coculture. Since Laurie et al proposed that nitroxoline delays the destruction of brain tissue, it could imply that the brain organoid will be more severely damaged. In addition, we intended to demonstrate the use of brain organoids as an alternative model to human brain tissue. Thus, we reason that the experimental design should be comparable to the study of Laurie et al. We finally decided to evaluate cell damage on day 4 post the coculture of human brain organoids with *B. mandrillaris*.

3. What would happen if the drug was administered to an already established infection rather than at the beginning of the coculture, which is what the clinical cases are all about?

**Response**: We do agree that drug administration after the infection would mimic the clinical setting of BAE more than our experiment design. As you kindly pointed out, we state the purpose of the brain organoid analysis after 4 days. Our work primarily aims to confirm the protective effect of nitroxoline in the context of human brain organoids. Therefore, we discuss the limitations of our study regarding the nonrelevant setting of drug exposure (lines 491-497, pages 23-24). We hope the purpose of our study is clear and experimental design can address a research question.

**3. Minor**:

3.1 In both the abstract and the author summary, the authors claim that the use of human cerebral organoids (helps) unravel the host response. I would not say this, because while this model is certainly important for understanding the mechanisms of amoeba pathogenicity, it lacks, as the authors themselves admit, the complexity of the host, especially the immune system.

**Response**:

You are right. We edited the abstract from “unravel the host response” to “important for understanding the mechanism of amoeba pathogenicity” since the brain organoid model still lacks of immune system.

The changes are in lines 38-39 (page 2) of the revised manuscript.

3.2 Lines 89-95. Text flow is a bit confusing.

**Response**: We modified the sentences to avoid confusion.

Original: The use of human cerebral organoids reveals the molecular mechanism underlying microcephaly caused by viral infections. Zika virus impairs early brain development, which can be prevented by adding interferon-beta.

Revised: Human cerebral organoids are useful for the study of mechanisms underlying virus-caused microcephaly. By using human brain organoid, Zika virus impairs early stage of brain organoid development, resulting in smaller size. Treatment of the brain organoid with interferon-b could ameliorate the zika-induced microcephaly.

We hope this text flow is understandable. The revised text is in lines 89-93 on page 5.

3.3 Line 80. Host-pathogen interaction“. Which pathogens?

**Response**: The pathogens are *Cryptococcus neoformans* and herpes simplex virus 1. We add these two pathogen names in lines 77-78 on page 4.

3.5 Line 76. should be “antiparasitics”.

**Response**: We replace it with “antiparasitics” (line 73 on page 4).

3.6 Line 141. When cysts appeared at less than 1%“ could you please explain?

**Response**:

We apologize for providing inaccurate information regarding the method of *B. mandrillaris* KM-20 strain culture. In the BM-3 medium, the trophozoites were routinely subcultured every 8 to 10 days but not 3-4 days as mentioned in the first version of the submitted manuscript. Moreover, the trophozoites rarely transform into cysts during the 8-10 days of culture.

The edited sentence of “the trophozoites were routinely subcultured every 8 to 10 days” is in line 139 on page 7.

3.7 Line 160 CO2. subscript

**Response**: Please refer to the line 159 (page 8).

3.8 Lines 210-211. Is there a reference for these markers?

**Response**: Yes, there are references. We add these references in line 210 (page10).

44. Li J, Yu C, Sun Y, et al. Serum ubiquitin C-terminal hydrolase L1 as a biomarker for traumatic brain injury: a systematic review and meta-analysis. Am J Emerg Med. 2015; 33(9): 1191-1196.

45. Jiang L, Wu Y, Zhang Y, et al. Effects of intraoperative lung-protective ventilation on clinical outcomes in patients with traumatic brain injury: a randomized controlled trial. BMC Anesthesiol. 2021; 21(1): 182.

46. Abdelhak A, Foschi M, Abu-Rumeileh S, et al. Blood GFAP as an emerging biomarker in brain and spinal cord disorders. Nat Rev Neurol. 2022; 18(3): 158-172.

3.9 Line 248. “FOXG1 is a transcription factor that of essential for forebrain development“ sentence structure error?

**Response**: You are right. We apologize for the error. Now, we add “FOXG1 is a transcription factor essential for forebrain development” in line 257 on page13.

3.10 Line 272. Can you explain how Fluo-4 works?

**Response**: Yes, we can. Fluo-4 is a fluorescence molecule that can permeate the cell membrane and bind calcium.

The revised portion: “After an hour of incubation and subsequent removal of excess Ca^2+^-binding Fluo-4, the green fluorescence of the 3D cell clusters was captured at 15-second intervals for a total of 20 minutes, yielding 1,199 images”, is in line 281 on page 14.

3.11 Lines 329-330. None of the chemokine (IL-8), inflammatory regulator (IL-1b and TNF-a), or immunostimulatory IFN-g transcript levels increased in the coculture.“ In fact, they have significantly decreased. Could you please explain why and what the consequences would be?

**Response:**

We add the explanation of cytokine gene expression in the discussion part as follows in lines 510-516 on page 24.

“A cerebral organoid is useful to study immune responses, particularly in Zika-induced microcephaly (32) IL-8, IL-1β, TNF-α, and IFN-γ reportedly induce intestinal inflammation caused by *Entamoeba histolytica*. They recruit neutrophils and macrophages to the site of amoebic invasion. Thus, a decrease of IL-8, IL-1β, TNF-α, and IFN-γ likely allows the amoeba to escape from inflammatory immune cells (56). Thus, reduction of IL-8, IL-1β, TNF-α, and IFN-γ imply the microenvironment devoid of immune responses, resulting in the survival of the *B. mandrillaris*. However, it needs to confirm cytokine release at the protein level and functional tests to conclude.”

There are new references added as well.

32. Krenn V, Bosone C, Burkard TR, Spanier J, Kalinke U, Calistri A, et al. Organoid modeling of Zika and herpes simplex virus 1 infections reveals virus-specific responses leading to microcephaly. Cell Stem Cell. 2021;28(8):1362-79.e7.

56. Uddin MJ, Leslie JL, Petri WA Jr. Host Protective Mechanisms to Intestinal Amebiasis. Trends Parasitol. 2021 Feb;37(2):165-175.

3.12 Lines 344-347. Please write which conditions (treated/untreated).

**Response**: we added “treated” in line 357 on page 18.

3.13 Fig. 2E. Why only showing treated sample?

**Response:**

Our initial purpose is to show the unique characteristics of nitroxoline-treated cerebral organoid, which is absent in the untreated cerebral organoid that co-culture with *B. mandrillaris*. To emphasize the specific characteristics, we add the untreated cerebral organoid co-cultured with the trophozoites.

More explanation was added in the results section (lines 365-370 one page 18).

“However, there were no trophozoites located in the hollow region of the brain organoids that were exposed to DMSO as vehicle control (S5 Fig).”

3.14 Lines 461-463. Please write how CCL7 is changed in your data.

**Response**:

Original = In addition, CCL7 is released from astrocytes and mediates neuroinflammation via activation of microglia.

Modified = In addition, CCL7 is upregulated in the cerebral organoids co-cultured with B. mandrillaris and reportedly released from astrocytes and mediates neuroinflammation via activation of microglia.

The change is in lines 504-505 on page 24.

3.15 Line 478. “Further validation with clinical samples from BAE patients has highlighted the utility of these biomarkers for early diagnosis and for studying response to treatment.” Is it true? Do you have a reference for this statement?

**Response**:

We apologize for the overstatement without reference. Thus, we edited the sentence. The new sentence: “Further validation with clinical samples from BAE patients may highlight the utility of these biomarkers for early diagnosis and for studying the response to treatment”, is added in line 529 on page 25.

3.16 Fig. S2 Please provide better description in legend: which time points, CMDFA/DiD staining explanation. You don't mention the middle panel and I don't see asterisks in the right panel.

**Response**:

We apologize for the insufficient description and lack of asterisks.

Here is the modified version of S2 Figure (772-773).

**S2 Fig.** **Coculture of** ***B. mandrillaris* trophozoites with human cerebral organoids.** (A) Representative fluorescence images of cerebral organoids cocultured with trophozoites (scale bars, 200 µm). The cerebral organoids and the trophozoites were differentially labelled using the protein-binding CMFDA (green) and lipid-binding DiD (magenta) fluorescent markers, respectively. The 60-day cerebral organoid was incubated with the CMFDA for 1 hour followed by the co-culture with the DiD-labelled trophozoites. **(B)** Microscopy images of cerebral organoids cultured with trophozoites. On the surface, the trophozoites had multiple cytoplasm protrusions with active movement (arrowheads, left panel). Two days later, there were many trophozoites located at a small focal break in the cerebral organoids (arrowheads, right panel). Some trophozoites remained elongated in shape (asterisks, right panel), while most were oval (arrowheads, right panel). Scale bars, 100 µm.

3.16 Transcriptomics: Shouldn't there be some more detailed data in the supplement?

**Response**:

We add a table of transcripts which are up-and down-regulated in Supplementary Table 1 (line 819) and the list of top 100 highly expressed genes in Supplementary Table 2 (line 821). A brief explanation of data is in lines 404-406 (page 20).

**Reviewer #2**: Study design and methods are generally appropriate and well described.
1. It is not clear why expression level for some of the genes of interest required quantitative PCR, while Illumina NGS and bioinformatics analysis was suitable for others, as described in lines 198-204.

**Response**:

We performed quantitative PCR to characterize cerebral organoids in terms of cell types and their maturity. This qPCR assay is essential as a quality control method of the brain organoid before using as a disease model.

By contrast, we used Illumina NGS and bioinformatics to examine any differentially transcribed genes in a non-specific manner. These transcriptional changes of cerebral organoids” allow us to compare types and levels of transcripts not only specific to cell types but also cover other genes involving in various functions such as cell structure, cell signaling pathways or innate immune responses.

We hope that our explanation would address your concern.

2. In the Figure 1 legend, line 298 refers to 'regions of interest (ROI) are outlined in the left panel'. This panel seems to have been omitted.

Response: We apologize for the mistake. Now, we added the representative image of the Fluo-4-labelling cerebral organoid in Fig. 1F.

Here, the description in the revised manuscript, “(F) Calcium influx and efflux of the cerebral organoids using Fluo-4 and live imaging for 20 minutes (regions of interest (ROIs) are outlined in the left panel) as measured by changes in fluorescence (arbitrary units). Three individual organoids were tested (n = 3).”

The change is in lines 305-308 on page 15.

3. In Figure 2, apparent degeneration of amoebae in the hollow interior of the treated cerebral organoids was shown, but not the corresponding image of untreated control organoids; so it is not clearly demonstrated that changes were due to treatment, as vacuolation could be a tissue-processing artefact. Also, what were the morphological changes induced by miltefosine - were they similar to those shown in 2D & 2E?

**Response**:

Thanks for pointing out the missing important data. In the revised manuscript, we show the microscopic image of the untreated cerebral organoid. To our observation, there was no trophozoite in the hollow interior of the untreated cerebral organoid. We provide the image of the untreated control as supplementary figure (S5 Fig.). The explanation of the Supplementary Figure (S5 Fig) is in lines 797-806 on page 34-35.

For miltefosine, we could not observe the vacuolated trophozoites in the hollow or inner layer of the cerebral organoid. Thus, it seems that the specific to the nitroxoline treatment. We provide the image of the miltefosine-treated cerebral organoid as a supplementary figure (S5 Fig. and its figure legend in lines 797-806). The explanation of the S5 figure is in lines 365-370 on page 18.

4. Miltefosine is recommended for the treatment of *B. mandrillaris* infection; it would be helpful to show the dose-response curve for miltefosine alongside or together with that of nitroxoline - it could be on the same graph since a log scale is used.

**Response**: We added the dose-response curve of miltefosine in the same curve of nitroxoline. Please refer to Fig. 2C.and the legend of Fig. 2 (lines 383-386 on page 18).

In the Fig. 2 legend, the meaning of arrows and arrowheads for 2D should also be specified here.

**Response**:

We edited the figure 2 legend in lines 388-393 as follow:

Here is the revised portion, “(D) H&E staining of cerebral organoids cocultured with trophozoites with and without nitroxoline (scale bars, 20 µm). In the DMSO-treated control (left panel), the trophozoites invaded the inner layer (arrowhead), while the meninge-like layer detached from the outermost layer (arrow). In the nitroxoline-treated samples (right panel), the outermost layer of the cerebral organoids remained intact (arrow). Some trophozoites had a large unstained space in the cytoplasm (arrowheads).”

Figure 3: 3B heatmap shows 6 columns of transcripts, but it is not specified which are the 2 controls and 2 tests (line 404 in the legend). For the non-expert reader, 3C is difficult to understand, likewise 3E - what is the difference between the left and right sides of the image? Although mentioned in the text, the legend for Fig 3 should also specify what 3D shows.

**Response**:

We apologize for missing the labels.

1) Fig. 3B was modified by adding “control” and “test”.
2) In Fig. 3C, the upper panel shows each cluster of genes in different ontogeny with different colors, while the lower panel indicates a “low to high level” of statistically significant differences as light yellow to dark red. We added this explanation in lines 432-434 of the figure legend (page 21).
3) In Fig. 3D, they lack labeling. We now added the proper labeling (lines 435-441 on page 21).

Reviewer #2: Discussion and conclusions are otherwise appropriate. Regarding line 452, in the present study it would have been interesting to investigate whether brain damage markers were reduced in nitroxoline-treated organoids, compared with untreated ones. Line 479 has relevance to this question.

**Response**:

We do agree with the suggestion and added the suggestion for further investigation using clinical samples of BAE patients. Here are the edited sentences in the discussion (lines 529-532 on page 25).

“Further validation with clinical samples from BAE patients may highlight the utility of these biomarkers for early diagnosis and for studying the response to treatment. For instance, reduction in levels of the brain damage markers should be investigated in the nitroxoline-treated cerebral organoids. Nevertheless, the scarcity of BAE has hindered such clinical validation.”

Reviewer #2: See suggestions in the Results review.
Line 35 or 111: show abbreviation iPSCs, as this is the first use.
Line 76: change to ...antiparasitics but are largely ineffective.
Line 146: show provenance of the drugs.
Line 201-202: repetition of information.
Line 248: delete 'that of'
Line 287: The data are the mean of triplicates of the relative expression level +-SD. Delete next sentence.
Line 344: Insert 'treated' in front of 'cerebral'.
Line 355: Replace 'lining' with 'covering'.
Line 358: Specify meaning of arrows in 2A(c).
Line 394: Remove comma after 'pathways'.
Line 424: Provide reference no.
Lines 557, 573, 654, 655: italicise scientific names.
Line 633: change to 'specimens'.

**Response**:

We highly appreciate for helping us to correct the typos. Please find our edits in the table.

| **Reviers’ suggestions** | **Modified** |
| --- | --- |
| Line 35 or 111: show abbreviation iPSCs, as this is the first use. | “human induced pluripotent stem cells (iPSCs)” (line 106) |
| Line 76: change to ...antiparasitics but are largely ineffective. | “Antiparasitics” (line 73) |
| Line 146: show provenance of the drugs. | “obtained from Dr. Christopher A Rice at Purdue University College of Veterinary Medicine” (lines 149-150) |
| Line 201-202: repetition of information. | Delete “Sequencing was performed on the Illumina NGS sequencing platform” (line 201) |
| Line 248: delete 'that of' | Delete “that of” (line 257) |
| Line 287: The data are the mean of triplicates of the relative expression level +-SD. Delete next sentence. | Delete “The data are the mean ± SD of technical triplicates.” (line 296) |
| Line 344: Insert 'treated' in front of 'cerebral'. | Add “treated” (line 357) |
| Line 355: Replace 'lining' with 'covering'. | “Covering” (line 375) |
| Line 358: Specify meaning of arrows in 2A(c). | “a trophozoite projected long pseudopodia proximal to neurons that were degenerated and necrotic (arrows in subpanel c and d) (line 378) |
| Line 394: Remove comma after 'pathways'. | Remove comma (line 421) |
| Line 424: Provide reference no. | Provide ref no. after Mondello et al. (51) (line 460) |
| Lines 557, 573, 654, 655: italicise scientific names. | Line 616, ref. No. 12 Liu et al., 2023 => *Balamuthia*  Line 631, ref. No. 18 Peng et al., 2022 => *Balamuthia*  Line 735, ref. No. 54 Parker et al., 2006 => *Drosophila*  Line 737, ref. No. 55 Zhu CC => *Drosophila* |
| Line 633: change to 'specimens'. | Line 701, Ref. no. 43 Win et al., 2019 |

Reviewer #2: Interesting, novel and generally well-written. GAE is an emerging disease and although rare, is usually fatal. Any promising advance in infection modelling and treatment is important.
**Response**: We agree that BAE modeling is really important. We do appreciate for your valuable and constructive comments.

Reviewer #3: The purpose of the study is clearly stated with a clear and testable hypothesis.
-The study design is appropriate to meet the stated objectives.
-Accurate statistical analysis was used to support the results.
-There are no concerns about meeting ethical or regulatory requirements.

Reviewer #3: The analysis presented matches the analysis plan.
-The results are presented clearly and completely.
-Figures (Tables, Visuals) are not of sufficient quality in terms of understandability. Readability and quality should be increased.

**Response**: We highly appreciate your suggestion to improve quality of our work. We submitted the high-resolution images in the revised manuscript.

Reviewer #3: -The results are supported by the data presented.
-Limitations of the analysis are not clearly defined.

**Response**: We explain the limitations of cerebral organoids in the revised manuscript (lines 491-497; 529-532).

Reviewer #3: First of all, I want to thank to all of the authors of the manuscript. Granulomatous amebic encephalitis, a central nervous system infection caused by Balamuthia mandrillaris, is a life-threatening infection. There are very few cases diagnosed and treated with this infection. There is a significant gap in both the treatment and diagnosis of this disease. Filling these gaps with the creation of such new models will offer new treatment and diagnostic options. This study is a good and comprehensive study. However, it would be better to increase the resolution of the figures to make them readable.

**Response**: The resolution of the submitted images was increased.

| **The revised Figure** | **Resolution (dpi?)** |
| --- | --- |
| Fig. 1 | 330 |
| Fig. 2 | 330 |
| Fig. 3 | 330 |
| Fig. 4 | 330 |
| S1 Fig. | 330 |
| S2 Fig. | 330 |
| S3 Fig. | 330 |
| S4 Fig. | 330 |
| S5 Fig. | 330 |
